# Impact of COVID-19 on HIV Testing Among AIDS Institute–Funded Providers in New York State–A Time Series Analysis

**DOI:** 10.1097/QAI.0000000000003109

**Published:** 2022-10-10

**Authors:** Thomas J. O'Grady, Yingchao Yuan, Julie M. Harris, Ronald J. Massaroni, John A. Fuller, James M. Tesoriero

**Affiliations:** New York State Department of Health, AIDS Institute, Albany, NY.

**Keywords:** HIV, HIV testing, COVID-19

## Abstract

New York State (NYS) was at the intersection of the HIV epidemic and coronavirus disease 2019 (COVID-19) pandemic leading to a disruption in HIV-preventive services. This study sought to determine the impact of the COVID-19 pandemic and mitigation efforts on HIV-testing trends in NYS among AIDS Institute (AI)-funded providers.

**Methods:**

We analyzed weekly testing data from the AI Reporting System from January 1, 2017, to June 27, 2021, to fit an interrupted time series model that predicted the expected number of HIV tests among AI-funded providers in NYS had the COVID-19 pandemic not occurred. The actual observed numbers of HIV testing that occurred from weeks beginning March 15, 2020, to June 30, 2021, were compared with the number of HIV tests predicted by the model.

**Results:**

In the absence of the COVID-19 pandemic, our model predicted that there would have been 45,605 HIV tests among AI-funded providers between the weeks beginning March 15, 2020, to June 27, 2021. We observed 20,742 HIV tests, representing a 54.5% reduction. We observed percent decreases of greater than 50% for HIV testing among AI-funded providers for New York City (52.9%) and rest of state (59.8%) regions, male (50.6%) and female (66.8%) genders, as well as Black (59.2%), Hispanic (52.8%), mixed race (57.5%), other (50.3%), and White (50.1%) race and ethnicities.

**Conclusion:**

HIV testing among AI-funded providers in NYS has declined substantially following the COVID-19 pandemic, reflecting decreased access to, and/or demand for, testing among persons at elevated risk for HIV. Initiatives to increase HIV testing and maintain access to HIV prevention services need to be explored following COVID-19.

## Abstract 2

Supplemental Digital Content is Available in the Text.

## INTRODUCTION

The Governor of the State of New York issued Executive Order “New York State (NYS) on PAUSE” on March 20, 2020, as a 10-point policy to ensure uniform safety for New Yorkers in response to the outbreak of coronavirus disease 2019 (COVID-19) caused by the novel severe acute respiratory syndrome coronavirus 2 (SARS-CoV-2).^1^ During this time, the Centers for Disease Control and Prevention additionally recommended individuals and groups practice social distancing to reduce exposure to SARS-CoV-2.^2^ A combination of the enacted policies and recommended guidelines to reduce exposures led to a subsequent decrease in access and use of preventive health care services.^3,4^ NYS has long been an epicenter for the HIV epidemic in the United States (US) and has the second-highest number of persons living with diagnosed HIV (PLWDH) in the US and the highest number of PLWDH per capita in the nation.^5^ NYS was also disproportionally affected in the early stages of the COVID-19 pandemic,^6^ providing NYS with a unique position to describe the intersection of these 2 large-scale public health concerns.

NYS formally announced a plan to end its AIDS epidemic in 2014 as a result of years of robust HIV policy and programming, strong state fiscal commitment, actively engaged community stakeholders, advances in medical care, favorable epidemiological conditions, and new HIV prevention tools such as pre-exposure prophylaxis (PrEP) for individuals at high risk of HIV infection.^7^ New York's plan outlined 3 core components to its end its AIDS epidemic agenda: (1) the identification of persons living with HIV who remain undiagnosed and linking those individuals to health care, (2) promoting health and preventing further transmission by maximizing viral suppression among PLWDH, and (3) facilitating access to PrEP and nonoccupational postexposure prophylaxis for high-risk individuals to facilitate maintaining a continued HIV-negative status.

The NYS Department of Health AIDS Institute (AI) funds community–based providers to deliver HIV testing services to persons at elevated risk for acquiring HIV, including those on PrEP. Before the start of the COVID-19 pandemic, HIV testing trends among AI-funded providers had been stable for many years. Shelter in place guidance, such as NYS on pause, and subsequent social distancing guidelines that were issued in response to COVID-19 resulted in a profound change to how health care was accessed due to health care providers closing their practices, switching to providing urgent care, or providing telehealth services.^8,9^ Changes to how health care professionals provided care and individual concerns regarding exposure to SARS CoV-2 likely impacted the provision of health care related to HIV preventive services. The objective of this study was to evaluate the impact of the COVID-19 pandemic on HIV testing among AI-funded providers in NYS.

## METHODS

This is a time series analysis of HIV testing in NYS among AI-funded providers following the COVID-19 pandemic as an unplanned natural public health intervention or natural experiment. This study followed all relevant Strengthening the Reporting of Observational Studies in Epidemiology reporting guidelines. This analysis used aggregate data on HIV testing without patient interaction. Therefore, no informed consent procedures were required, and the study received an exempt determination from the NYS Department of Health Institutional Review Board.

### Data Source

We analyzed data from the AI Reporting System (AIRS) database from January 2017 through June 2021. The AIRS database is a comprehensive client and service/encounter reporting application intended to support a broad range of provider types, from primary care clinics to multiservice community-based organizations.^10^ AIRS functions as a distributed system installed in over 200 individual agencies for internal data management and reporting but is capable of generating client-level data for state and regional databases. AIRS captures HIV testing data among entities funded by the AI. Funded providers include hospitals, community health centers, and federally qualified health centers located in high HIV seroprevalence areas, local health departments, syringe exchange programs, and other community-based organizations serving low-income persons, including those at elevated risk for HIV and other sexually transmitted infections. Testing occurs in medical settings, in community-based organizations, and in supported community venues such as drop-in centers, homeless shelters, and mobile vans.

### Outcomes

We identified HIV testing events captured in the AIRS database. While the total number of HIV tests conducted in NYS is unknown, providers reporting in AIRS have historically diagnosed approximately 6%–10% of all new HIV diagnoses occurring in NYS annually. The primary outcome measure in this study was the number of HIV tests enumerated as the weekly cumulative number of HIV tests among AI-funded providers throughout the study period. The outcome measures were reported overall and by demographic information including region (New York City [NYC] and rest of state [ROS]), gender (male, female, and transgender), and race (Black, Hispanic, mixed race, others, and White).

### Statistical Analysis

Weekly HIV testing data before and after the start of the COVID-19 pandemic were modeled as an interrupted time series using an autoregressive, integrated, moving average model adjusting for seasonality.^11–13^ This allowed for the modeling of a sequence of repeated weekly observations, HIV testing, which was interrupted by an event, the COVID-19 pandemic, which occurs at a specific known point in time. The COVID-19 impact was evaluated through the comparison of the expected trend in HIV testing that would have occurred in the absence of the pandemic with the observed change in HIV testing that occurred in the time period after the start of the pandemic. The time series model was used to project HIV testing numbers for the period of weeks beginning March 15, 2020, through June 27, 2021, the COVID-19 pandemic not occurred. A comparison of predicted HIV tests with observed HIV test counts during the weeks beginning March 15, 2020, through June 27, 2021, using the percent difference ([Projected Weekly HIV Tests—Expected Weekly HIV Tests]/Projected Weekly HIV Tests X 100%) to measure the COVID-19 impact. In addition, we graphed the projected number of weekly HIV tests for weeks beginning March 15, 2020, through June 27, 2021, COVID-19 not occurred, and the 95% confidence intervals (CIs) of the projected values and compared the plots of expected values of weekly HIV tests to these values. Finally, the estimated values of projected weekly HIV tests during the COVID-19 period were also stratified by patient demographic characteristics. All analyses were performed using SAS v9.4 [SAS Cary Institute Inc., Cary, NC].

## RESULTS

**FIGURE 1.**
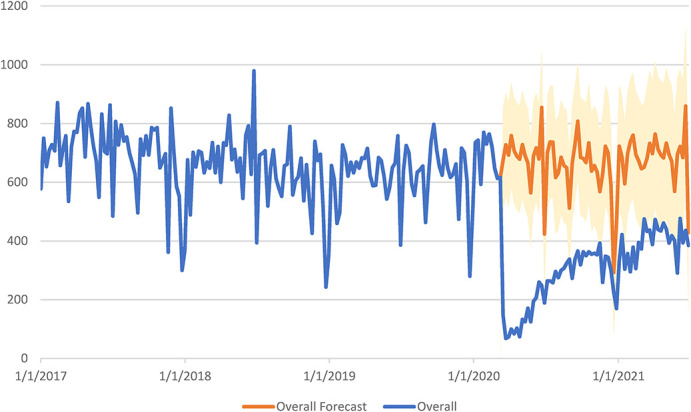
Total HIV testing among AI-funded providers in NYS from January 1, 2017, through June 27, 2021^a,b^. ^a^Weekly HIV tests are presented as counts in NYS for the dates January 1, 2017, through June 27, 2021. The blue line represents the total counts of actual weekly HIV tests that occurred among AI-funded providers. The orange line represents the forecasted total weekly HIV tests that would have occurred among AI-funded providers if COVID-19 had not occurred, and the shaded orange region represents the 95% confidence limits for the forecasted total HIV-testing projections. ^b^Interrupted time series models were created with the timing of the COVID-19 pandemic as the intervention. We used a technique developed by Box and Taio based on the Box–Jenkins ARIMA time series model to model and then project the forecasted weekly counts of HIV testing and the corresponding 95% confidence limits of these projections during the post–COVID-19 period (March 15, 2020, through June 27, 2021) for total HIV testing among AI-funded providers. ARIMA, autoregressive, integrated, moving average

**FIGURE 2.**
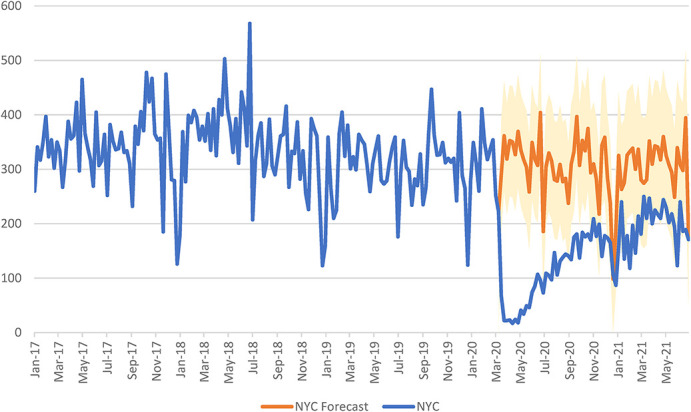
Total HIV testing among AI-funded providers in NYC from January 1, 2017, through June 27, 2021^a,b^. ^a^Weekly HIV tests are presented as counts in NYC for the dates January 1, 2017, through June 27, 2021. The blue line represents the total counts of actual weekly HIV tests that occurred among AI-funded providers. The orange line represents the forecasted total weekly HIV tests that would have occurred among AI-funded providers if COVID-19 had not occurred, and the shaded orange region represents the 95% confidence limits for the forecasted total HIV-testing projections. ^b^Interrupted time series models were created with the timing of the COVID-19 pandemic as the intervention. We used a technique developed by Box and Taio based on the Box–Jenkins ARIMA time series model to model and then project the forecasted weekly counts of HIV testing and the corresponding 95% confidence limits of these projections during the post–COVID-19 period (March 15, 2020, through June 27, 2021) for total HIV testing among AI-funded providers. ARIMA, autoregressive, integrated, moving average

**FIGURE 3.**
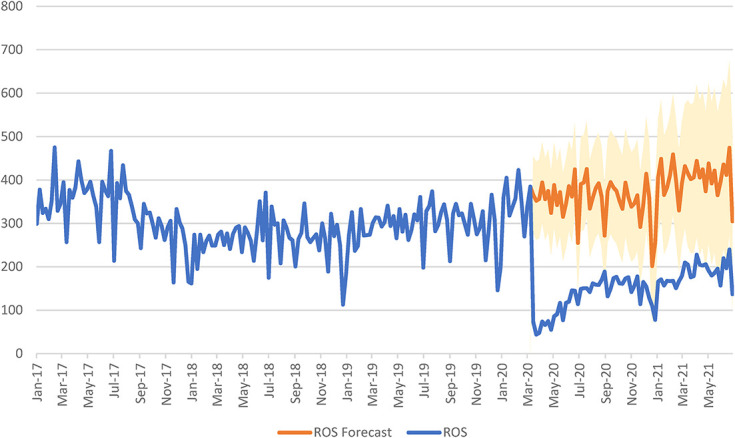
Total HIV testing among AI-funded providers in ROS (NYS, excluding NYC) from January 1, 2017, through June 27, 2021^a,b^. ^a^Weekly HIV tests are presented as counts in ROS (NYS, excluding NYC) for the dates January 1, 2017, through June 27, 2021. The blue line represents the total counts of actual weekly HIV tests that occurred among AI-funded providers. The orange line represents the forecasted total weekly HIV tests that would have occurred among AI-funded providers if COVID-19 had not occurred, and the shaded orange region represents the 95% confidence limits for the forecasted total HIV-testing projections. ^b^Interrupted time series models were created with the timing of the COVID-19 pandemic as the intervention. We used a technique developed by Box and Taio based on the Box–Jenkins ARIMA time series model to model and then project the forecasted weekly counts of HIV testing and the corresponding 95% confidence limits of these projections during the post–COVID-19 period (March 15, 2020, through June 27, 2021) for total HIV testing among AI-funded providers. ARIMA, autoregressive, integrated, moving average

The observed weekly number of HIV tests for weeks beginning January 1, 2017, through June 27, 2021, and the projected trends in HIV testing from the autoregressive, integrated, moving average models for weeks beginning March 15, 2020, through June 27, 2021, overall and for NYC and ROS (NYS, excluding NYC) are shown in Figures 1–3. All time series models for individual demographic characteristics are included in Figures S1–9, Supplemental Digital Content, http://links.lww.com/QAI/B986. The interrupted time series model projected an estimate of 45,605 total HIV tests among AI-funded providers during the weeks beginning March 15, 2020, through June 27, 2021, in the absence of the COVID-19 pandemic. The observed number of actual tests that occurred during this same time was 20,742. The percent reduction in number of HIV tests among AI-funded providers during the study period was 54.5% (Table 1). Figure 1 displays a graph of both the observed and projected numbers of weekly HIV tests (with 95% CIs) among AI-funded providers. Figure 1 demonstrates that the largest drop in HIV testing occurred right at the beginning of the COVID-19 pandemic and that there was a gradual upward trend in HIV testing toward pre-COVID-19 pandemic levels. Although there was an observed rebound in HIV tests among AI-funded providers, the number of tests remained significantly lower than the projected number of HIV tests that were expected had the COVID-19 pandemic not occurred.

**TABLE 1.** Observed and Expected Number of HIV Tests Among AI-Funded Providers and Predicted Percent Reduction From March 15, 2020, Through June 30, 2021, Stratified by Demographic Characteristics and AIRS Database

Characteristic	HIV Testing		
	Observed No.	Expected No.†	Percent Reduction (95% CI)
Total	20,742	45,605	54.5 (54.4 to 54.7)
Region			
NYC	9814	20,840	52.9 (52.6 to 53.2)
ROS	10,219	25,445	59.8 (59.6 to 60.0)
Gender			
Man	14,724	29,796	50.6 (50.4 to 50.8)
Woman	4370	13,150	66.8 (66.4 to 67.1)
Transgender	889	1390	13.9 (10.3 to 17.3)
Age group (yr)			
Younger than 50 years	17,878	38,353	53.4 (53.2 to 53.6)
50+	2864	7749	63.0 (62.7 to 63.4)
Race/ethnicity*			
Black	5680	13,914	59.2 (58.9 to 59.5)
Hispanic	6397	13,558	52.8 (52.5 to 53.2)
Mixed race	435	457	57.5 (55.9 to 59.4)
Others	51	2044	50.3 (49.1 to 51.4)
White	79	14,409	50.1 (49.7 to 50.4)

* Includes Asian, Native Hawaiian/Pacific Islander, and Alaskan Native/Native American.

† Expected number and predicted percent reduction during March 15, 2020–June 30, 2021 were estimated from interrupted time series models fit using a technique developed by Box and Taio based on the Box–Jenkins ARIMA time series model to model and then project the forecasted weekly counts of HIV testing and the corresponding 95% confidence limits of these projections during the post–COVID-19 period (March 15, 2020, through June 27, 2021) for total HIV testing among AI-funded providers.

ARIMA, autoregressive, integrated, moving average.

Table 1 provides the percent reduction in the number of HIV tests among AI-funded providers by region, gender, race, and ethnicity. There was a 52.9% and 59.8% reduction in HIV tests among AI-funded providers in NYC and ROS, respectively. Figure 2 shows that HIV testing numbers began to rebound after a large decrease in HIV tests at the start of the COVID-19 in NYC. Although there is still a reduced amount of testing in NYC, by the end of the time series, the numbers are within the projected 95% CIs. Figure 3 demonstrates that after the large decrease in HIV testing at the beginning of COVID-19 in ROS, there is some increase in the number of weekly HIV tests, but these numbers never return to their prepandemic levels and they never cross into the 95% CIs of the projected number of tests.

There was a decrease in the number of HIV tests among AI-funded providers for men (50.6%), women (66.8%), and transgender (13.9%) individuals (Table 1). The largest drop in the number of weekly HIV tests among AI-funded providers occurred at the beginning of the pandemic for men, and although numbers increased thereafter, the number of observed HIV tests among men remained outside the 95% CIs for the projected number of HIV tests during the study period (see Figure S1, Supplemental Digital Content, http://links.lww.com/QAI/B986). For women and transgender individuals, the number of observed weekly HIV tests among AI-funded providers returned to prepandemic projected levels by the end of the study period (see Figures S2 and S3, Supplemental Digital Content, http://links.lww.com/QAI/B986). There was a decrease in the number of observed HIV tests among AI-funded providers for Black (59.2%), Hispanic (52.8%), mixed race (57.7%), others (50.2%), and White (50.1%) during the COVID-19 study period. The observed numbers of weekly HIV tests remained lower for only individuals who were of black race and ethnicity for the duration of the study period (see Figure S4, Supplemental Digital Content, http://links.lww.com/QAI/B986). Despite large drops in the observed number of weekly HIV tests for other race and ethnicity groups, the number of weekly HIV tests increased and returned to within the 95% CIs of the projected values by the end of the COVID-19 study period.

**FIGURE 4.**
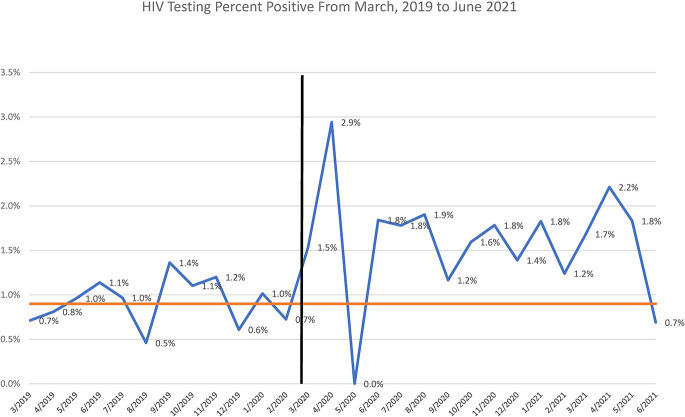
Percent positivity of HIV testing among AI-funded providers from March 2019 to June 2021. The orange line represents that average pre-COVID percent positivity of 0.9% for reference. The blue line represents the actual percent positivity over time.

Figure 4 displays the percentage of positive HIV tests over time. A clear increase in seropositivity is observable immediately after the start of the COVID-19 pandemic, with mean seropositivity increasing from 0.9% across the pre–COVID-19 months to 1.8% across the post–COVID-19 months. Owing to the large decrease in testing volume, the total number of HIV-positive tests were still higher across the pre–COVID-19 timeframe (14/month vs. 7/month) notwithstanding the higher seropositivity realized post-COVID-19.

## DISCUSSION

The number of HIV tests among AI-funded providers decreased markedly, by 54.5%, in NYS after the start of the COVID-19 pandemic. Although there was some rebound in the number of weekly HIV tests that occurred, the levels did not return to their pre-COVID levels in the 15-month follow-up period after the start of the pandemic. Our findings of a substantial initial decrease in HIV testing followed by a slight increase mirror the reports of an initial 68%–97% reduction in HIV tests during the state-at-home periods of 4 United States (US) municipalities that were followed by a slight increase but sustained 11%–54% decrease in HIV during transitory periods.^14^

It is difficult to determine exactly what is driving the decrease in HIV testing among AI-funded providers in NYS. The widespread stay-at-home orders that were enacted to control the spread of the SARS-CoV-2 virus was multifactorial in their potential for disruption. It has been reported that nonemergent outpatient clinical services were limited, providers shifted their practices to telehealth, laboratories closed or had limited ability to focus on non–COVID-19 activities, and physical spaces were modified in ways that reduced capacity and focused on treating patients with COVID-19.^15,16^ Patients may have avoided seeking medical services out of fear related to COVID-19 exposure at the clinic or by public transportation.^17^ Some patients may have also placed lower emphasis on seeking care due to competing needs related to the pandemic such as navigating unemployment and food insecurity.^18^ There is additional evidence that individuals with HIV may have been more likely to initiate care in the emergency department (ED) after the start of the COVID-19 pandemic.^19^ HIV testing in EDs may offer an opportunity to capture those individuals not picked up by traditional testing programs as the pandemic, and its effects continue, and as HIV testing levels continued below pre-COVID levels for the duration of our analysis. Mail-out HIV and sexually transmitted infection testing were readily used during a pilot in Washington, District of Columbia, during the COVID-19 pandemic indicating demand for HIV testing remained in high-priority demographic areas.^20^

The higher observed seropositivity rate during the post–COVID-19 period may indicate that those at higher-than-usual risk (including those with symptoms or illness) were more likely to seek testing during COVID-19. Importantly, the higher seropositivity post–COVID-19 was not enough to offset the impact of reduced testing, as the total number of persons testing positive was lower in the post–COVID-19 months. This finding is consistent across NYS overall, as the state experienced a decrease in the number of new HIV diagnoses (−18.8%) and new AIDS diagnoses (−22.3%) during 2020.^21^ Importantly, these decreases greatly exceeded historical annual decreases in new diagnoses being realized in NYS. A decrease in the number of new HIV diagnoses can lead to an increased risk of transmission opportunities among those unaware of their HIV status. Unfortunately, it is not possible to predict the true impact of reduced HIV testing on the HIV epidemic in NYS at this point, however, because risk behavior during COVID-19 may have also shifted. For example, 20% of PrEP using MSM surveyed in the Southern United States reported a decrease in all sexual behaviors from February to April of 2020; however, this decrease was followed by an increase from April to June.^22^ An Australian study indicated that although individuals who discontinued PrEP were less likely to report HIV testing, these individuals also were less likely to report casual sex partners indicating that the decrease in PrEP use observed among this group may coincide with a potential decrease in sexual activity.^23^ Sharp increases in gonorrhea were reported in NYS during COVID, with a 40% increase observed outside NYC and local counties reporting increases of more than 200%, indicating that there was still likely a high level of sexual activity occurring during this time period.^24^

Considering the decreases in HIV testing observed among AI-funded providers more than a year after the start of the COVID-19 pandemic, it is important to envision what the future of HIV testing looks like in NYS in the post–COVID-19 era. An emphasis on home test kits may be a useful way to reach those individuals and municipalities that may have limited access to health care services because of COVID-19.^20,25^ In addition, HIV screening volumes in the ED at the University of Chicago Medical Center remained stable despite a 49% reduction in testing from January 1 to April 30, 2020, in the Expanded HIV Testing and Linkage to Care Program, which represents 13 testing sites in Chicago's south and west sides.^26^ The authors further recommend that HIV testing should be incorporated with COVID-19 testing as patients with symptoms of acute HIV may present for COVID-19 testing. The linkage of COVID-19 testing with HIV testing is an important area for further investigation. Patients with acute HIV infection comprised 26.1% of new diagnoses in the University of Chicago Medical Center ED, indicating that patients with acute HIV infection may be more likely to present to the ED for screening. Modeling suggests that incorporating or linking HIV screening to COVID-19 testing in the ED setting would reduce HIV incidence and health care costs.^27^

### LIMITATIONS

The early and severe arrival of the COVID-19 pandemic in NYS may limit the generalizability of our results to other US jurisdictions. Also, limiting generalizability is the fact that our data represent HIV testing patterns among at-risk persons reached through AI-funded providers and not HIV testing patterns among New Yorkers more generally. Provider-specific differences in populations served and/or in the ability to resume HIV testing post-COVID could contribute to observed differences in COVID-19 impact across demographic categories. Our post–COVID-19 timeframe is relatively short, and more research is needed to understand the longer-term impact of the COVID-19 pandemic on HIV testing patterns. Finally, these data do not permit an understanding of the mechanism(s) by which the COVID-19 pandemic served to decrease HIV testing or an understanding of how possible changes in HIV risk-related behavior during the pandemic may have altered the real or perceived need for HIV testing among at-risk New Yorkers.

## CONCLUSIONS

Despite the noted limitations to our study, the impact of COVID-19 on HIV testing in NYS is substantial and warrants further investigation. A large and prolonged drop in HIV testing likely corresponds to an increase in the number of people who do not know their status. The implications of this study are missed opportunities to link and retain those with HIV to care to prevent further transmission and to facilitate PrEP access to help keep high-risk persons HIV negative.

## Supplementary Material

SUPPLEMENTARY MATERIAL
