# Supplementary material for: Impact of COVID-19 on HIV Testing Among AIDS Institute–Funded Providers in New York State–A Time Series Analysis
Source: J Acquir Immune Defic Syndr. 2022 Oct 10;92(1):27–33. doi: 10.1097/QAI.0000000000003109 (PMC9722363; doi:10.1097/QAI.0000000000003109)
Supplement: SUPPLEMENTARY MATERIAL [file qai-92-27-s001.docx]

Supplemental Figure 1: Total HIV Testing Among AIDS Institute Funded Providers in New York State for Male Gender from January 1, 2017 through June 27, 2021^a,b^

a Weekly HIV tests are presented as counts in New York State Male individuals for the dates 1/1/2017 through 6/27/2021. The blue line represents the total counts of actual weekly HIV tests that occurred among AIDS Institute funded providers. The orange line represents the forecasted total weekly HIV tests that would have occurred among AIDS Institute funded providers if COVID-19 had not occurred, and the shaded orange region represents the 95% confidence limits for the forecasted total HIV testing projections.

b. Interrupted time-series models were created with the timing of the COVID-19 pandemic as the intervention. We utilized a technique developed by Box and Taio based on the Box-Jenkins autoregressive, integrated, moving average (ARIMA) time-series model to model and then project the forecasted weekly counts of HIV testing and the corresponding 95% confidence limits of these projections during the post -COVID-19 period (3/15/2020 through 6/27/2021) for total HIV testing among AIDS Institute funded providers.

Supplemental Figure 2: Total HIV Testing Among AIDS Institute Funded Providers in New York State for Female Gender from January 1, 2017 through June 27, 2021^a,b^

a Weekly HIV tests are presented as counts in New York State for Female individuals for the dates 1/1/2017 through 6/27/2021. The blue line represents the total counts of actual weekly HIV tests that occurred among AIDS Institute funded providers. The orange line represents the forecasted total weekly HIV tests that would have occurred among AIDS Institute funded providers if COVID-19 had not occurred, and the shaded orange region represents the 95% confidence limits for the forecasted total HIV testing projections.

b. Interrupted time-series models were created with the timing of the COVID-19 pandemic as the intervention. We utilized a technique developed by Box and Taio based on the Box-Jenkins autoregressive, integrated, moving average (ARIMA) time-series model to model and then project the forecasted weekly counts of HIV testing and the corresponding 95% confidence limits of these projections during the post -COVID-19 period (3/15/2020 through 6/27/2021) for total HIV testing among AIDS Institute funded providers.

Supplemental Figure 3: Total HIV Testing Among AIDS Institute Funded Providers in New York State for Transender for January 1, 2017 through June 27, 2021^a,b^

a Weekly HIV tests are presented as counts in New York State for Transgender individuals for the dates 1/1/2017 through 6/27/2021. The blue line represents the total counts of actual weekly HIV tests that occurred among AIDS Institute funded providers. The orange line represents the forecasted total weekly HIV tests that would have occurred among AIDS Institute funded providers if COVID-19 had not occurred, and the shaded orange region represents the 95% confidence limits for the forecasted total HIV testing projections.

b. Interrupted time-series models were created with the timing of the COVID-19 pandemic as the intervention. We utilized a technique developed by Box and Taio based on the Box-Jenkins autoregressive, integrated, moving average (ARIMA) time-series model to model and then project the forecasted weekly counts of HIV testing and the corresponding 95% confidence limits of these projections during the post -COVID-19 period (3/15/2020 through 6/27/2021) for total HIV testing among AIDS Institute funded providers.

Supplemental Figure 4: Total HIV Testing Among AIDS Institute Funded Providers in New York State for Black/African American Race/Ethnicity for January 1, 2017 through June 27, 2021^a,b^

a Weekly HIV tests are presented as counts in New York State for Black/African American individuals for the dates 1/1/2017 through 6/27/2021. The blue line represents the total counts of actual weekly HIV tests that occurred among AIDS Institute funded providers. The orange line represents the forecasted total weekly HIV tests that would have occurred among AIDS Institute funded providers if COVID-19 had not occurred, and the shaded orange region represents the 95% confidence limits for the forecasted total HIV testing projections.

b. Interrupted time-series models were created with the timing of the COVID-19 pandemic as the intervention. We utilized a technique developed by Box and Taio based on the Box-Jenkins autoregressive, integrated, moving average (ARIMA) time-series model to model and then project the forecasted weekly counts of HIV testing and the corresponding 95% confidence limits of these projections during the post -COVID-19 period (3/15/2020 through 6/27/2021) for total HIV testing among AIDS Institute funded providers.

Supplemental Figure 5: Total HIV Testing Among AIDS Institute Funded Providers in New York State for Hispanic Race/Ethnicity for January 1, 2017 through June 27, 2021^a,b^

a Weekly HIV tests are presented as counts in New York State for Hispanic individuals for the dates 1/1/2017 through 6/27/2021. The blue line represents the total counts of actual weekly HIV tests that occurred among AIDS Institute funded providers. The orange line represents the forecasted total weekly HIV tests that would have occurred among AIDS Institute funded providers if COVID-19 had not occurred, and the shaded orange region represents the 95% confidence limits for the forecasted total HIV testing projections.

b. Interrupted time-series models were created with the timing of the COVID-19 pandemic as the intervention. We utilized a technique developed by Box and Taio based on the Box-Jenkins autoregressive, integrated, moving average (ARIMA) time-series model to model and then project the forecasted weekly counts of HIV testing and the corresponding 95% confidence limits of these projections during the post -COVID-19 period (3/15/2020 through 6/27/2021) for total HIV testing among AIDS Institute funded providers.

Supplemental Figure 6: Total HIV Testing Among AIDS Institute Funded Providers in New York State for Multi-Race/Ethnicity for January 1, 2017 through June 27, 2021^a,b^

a Weekly HIV tests are presented as counts in New York State for Multi-race/ethnicity individuals for the dates 1/1/2017 through 6/27/2021. The blue line represents the total counts of actual weekly HIV tests that occurred among AIDS Institute funded providers. The orange line represents the forecasted total weekly HIV tests that would have occurred among AIDS Institute funded providers if COVID-19 had not occurred, and the shaded orange region represents the 95% confidence limits for the forecasted total HIV testing projections.

b. Interrupted time-series models were created with the timing of the COVID-19 pandemic as the intervention. We utilized a technique developed by Box and Taio based on the Box-Jenkins autoregressive, integrated, moving average (ARIMA) time-series model to model and then project the forecasted weekly counts of HIV testing and the corresponding 95% confidence limits of these projections during the post -COVID-19 period (3/15/2020 through 6/27/2021) for total HIV testing among AIDS Institute funded providers.

Supplemental Figure 7: Total HIV Testing Among AIDS Institute Funded Providers in New York State for White Race/Ethnicity for January 1, 2017 through June 27, 2021^a,b^

a Weekly HIV tests are presented as counts in New York State for White race/ethnicity individuals for the dates 1/1/2017 through 6/27/2021. The blue line represents the total counts of actual weekly HIV tests that occurred among AIDS Institute funded providers. The orange line represents the forecasted total weekly HIV tests that would have occurred among AIDS Institute funded providers if COVID-19 had not occurred, and the shaded orange region represents the 95% confidence limits for the forecasted total HIV testing projections.

b. Interrupted time-series models were created with the timing of the COVID-19 pandemic as the intervention. We utilized a technique developed by Box and Taio based on the Box-Jenkins autoregressive, integrated, moving average (ARIMA) time-series model to model and then project the forecasted weekly counts of HIV testing and the corresponding 95% confidence limits of these projections during the post -COVID-19 period (3/15/2020 through 6/27/2021) for total HIV testing among AIDS Institute funded providers.

Supplemental Figure 8: Total HIV Testing Among AIDS Institute Funded Providers in New York State for Age Under 50 for January 1, 2017 through June 27, 2021^a,b^

a Weekly HIV tests are presented as counts in New York State for age under 50 individuals for the dates 1/1/2017 through 6/27/2021. The blue line represents the total counts of actual weekly HIV tests that occurred among AIDS Institute funded providers. The orange line represents the forecasted total weekly HIV tests that would have occurred among AIDS Institute funded providers if COVID-19 had not occurred, and the shaded orange region represents the 95% confidence limits for the forecasted total HIV testing projections.

b. Interrupted time-series models were created with the timing of the COVID-19 pandemic as the intervention. We utilized a technique developed by Box and Taio based on the Box-Jenkins autoregressive, integrated, moving average (ARIMA) time-series model to model and then project the forecasted weekly counts of HIV testing and the corresponding 95% confidence limits of these projections during the post -COVID-19 period (3/15/2020 through 6/27/2021) for total HIV testing among AIDS Institute funded providers.

Supplemental Figure 9: Total HIV Testing Among AIDS Institute Funded Providers in New York State for Age 50+ for January 1, 2017 through June 27, 2021^a,b^

a Weekly HIV tests are presented as counts in New York State for age 50+ individuals for the dates 1/1/2017 through 6/27/2021. The blue line represents the total counts of actual weekly HIV tests that occurred among AIDS Institute funded providers. The orange line represents the forecasted total weekly HIV tests that would have occurred among AIDS Institute funded providers if COVID-19 had not occurred, and the shaded orange region represents the 95% confidence limits for the forecasted total HIV testing projections.

b. Interrupted time-series models were created with the timing of the COVID-19 pandemic as the intervention. We utilized a technique developed by Box and Taio based on the Box-Jenkins autoregressive, integrated, moving average (ARIMA) time-series model to model and then project the forecasted weekly counts of HIV testing and the corresponding 95% confidence limits of these projections during the post -COVID-19 period (3/15/2020 through 6/27/2021) for total HIV testing among AIDS Institute funded providers.
